# Synthesis and Preliminary Biological Evaluations of Fluorescent or ^149^Promethium Labeled Trastuzumab-Polyethylenimine

**DOI:** 10.3390/biomedicines4010001

**Published:** 2015-12-30

**Authors:** Jonathan Fitzsimmons, Tapan Nayak, Cathy Cutler, Robert Atcher

**Affiliations:** 1 Chemistry, Life, and Earth Sciences Directorate, Los Alamos National Laboratory, Los Alamos, NM 87545, USA; ratcher@lanl.gov; 2 Department of Cell Biology and Physiology, School of Medicine, University of New Mexico Health Science Center, Albuquerque, NM 87131, USA; tapann@gmail.com; 3 College of Pharmacy, Radiopharmaceutical Sciences Program, University of New Mexico Health Science Center, Albuquerque, NM 87131, USA; 4 University of Missouri Research Reactor (MURR), University of Missouri-Columbia, Columbia MO 65211, USA; ccutler@bnl.gov

**Keywords:** Promethium-149, Trastuzumab, polyethylenimine, radiotherapy, targeted therapy, Actinium-225

## Abstract

Background: Radioimmunotherapy utilize a targeting antibody coupled to a therapeutic isotope to target and treat a tumor or disease. In this study we examine the synthesis and cell binding of a polymer scaffold containing a radiotherapeutic isotope and a targeting antibody. Methods: The multistep synthesis of a fluorescent or ^149^Promethium-labeled Trastuzumab-polyethyleneimine (PEI), Trastuzumab, or PEI is described. *In vitro* uptake, internalization and/or the binding affinity to the Her2/neu expressing human breast adenocarcinoma SKBr3 cells was investigated with the labeled compounds. Results: Fluorescent-labeled Trastuzumab-PEI was internalized more into cells at 2 and 18 h than fluorescent-labeled Trastuzumab or PEI. The fluorescent-labeled Trastuzumab was concentrated on the cell surface at 2 and 18 h and the labeled PEI had minimal uptake. DOTA-PEI was prepared and contained an average of 16 chelates per PEI; the compound was radio-labeled with ^149^Promethium and conjugated to Trastuzumab. The purified ^149^Pm-DOTA-PEI-Trastuzumab had a radiochemical purity of 96.7% and a specific activity of 0.118 TBq/g. The compound demonstrated a dissociation constant for the Her2/neu receptor of 20.30 ± 6.91 nM. Conclusion: The results indicate the DOTA-PEI-Trastuzumab compound has potential as a targeted therapeutic carrier, and future *in vivo* studies should be performed.

## 1. Introduction

The development of personalized cancer treatment has resulted in an increase in research of radioimmunotherapy agents [1]. These tumor specific agents are composed of a targeting antibody coupled to a therapeutic radioisotope and are able to deliver cytotoxic radiation to the targeted cells. Approximately 30% of breast cancer patients have tumors that have amplification and over expression of the Her2/neu receptor [2]. The expression of the receptor is highly correlated with aggressive forms of the disease, and the HER2 receptor expression is similar between the primary tumor and the corresponding secondary metastases [3,4]. The humanized monoclonal antibody, Trastuzumab, has been used in combination with radiation and chemotherapy for treatment of Her2/neu-positive breast cancer. Trastuzumab binds to the HER2 protein and modulates multiple signaling targets and pathways which lead to cell cycle G1 arrest and growth inhibition [5]. The expression of the HER2/neu receptor is an attractive target for imaging and therapy applications. A molecule that had affinity for the receptor would deliver its payload to the primary tumor and the corresponding metastases could be a benefit to cancer treatment.

The ^225^Actinium (^225^Ac) decay chain has been called an atomic scale *in vivo* alpha particle generator with the capability to kill cells [6]. The ^225^Ac decay chain has four alpha and two beta particle emissions and can deliver 28 MeV for each atom of ^225^Ac that decays to stable ^209^Bismuth. This high level of radiation can be highly lethal to cells. The ^225^Ac has a half-life of 10 days making it suitable for antibody targeting *in vivo* [7]. The polymer scaffold; polyethylenimine (PEI), was modified with a primary chelator for Actinium-225 [(1,4,7,10-tetraazacyclo-dodecane-1,4,7,10-tetrayl) tetraacetic acid; (DOTA)], and secondary chelators to capture the recoiling daughters [8]. This molecule was able to capture greater than 50% of the ^225^Ac daughters out to 150 h. In subsequent studies a pre-labeling approach was developed to potentially incorporate ^225^Ac into a targeted PEI [9]. No *in vitro* studies were performed with the targeted PEI, so this research examined the *in vitro* targeting of Trastuzumab-PEI.

PEI is a branched polymer that is internalize into cells and used for gene delivery [10], so the molecule could be ideal for delivery of therapeutic radionuclides to the inside of diseased cells. PEI has been shown to act as a proton sponge and cause enhanced endosomal chloride accumulation and osmotic swelling/lysis [11]. A 60 kDa PEI has approximately 1000 amines with 750 of the amines as either secondary or primary and provides a large number of reactive sites for targeting groups, imaging probes, and/or chelators with diagnostic or therapeutic isotopes. The highly branched PEI has been shown to be resistant to radiation damage [12] and was chosen as the scaffold to attach Trastuzumab for targeting and radio- therapeutic isotopes. Branched PEI is not degraded *in vivo* and is considered highly toxic [13], and some researchers have used PEI in copolymers to reduce toxicity during gene delivery [14]. The patient dose for ^225^Ac therapy has been estimated at 1480 kBq (40 µCi) (3.06 × 10^−12^ mol) [15], so small mass amounts of PEI could be used for radiotherapy applications and be below toxic levels [16].

To perform the *in vitro* studies we substituted Promethium-149 (^149^Pm) for ^225^Ac. Both radionuclides have a 3+ oxidation state and are coordinated by DOTA derivatives with similar serum stability. An ^225^Ac DOTA complex had a serum stability of ~90% at 10 days, and the ^149^Pm DO3A-AmideBBN complex had a serum stability of >94% at five days [8,17]. ^225^Ac has no stable isotopes, and ^149^Pm can be produced with high specific activity (purity) [18], so the purity of both radionuclides. ^149^Pm has a half-life of 2.21 days, and the radionuclide is a dual emission isotope with a beta maximum energy of 1.07 MeV (95.9%) for therapy, and a gamma ray at 285.9 keV (3%). The gamma ray allows imaging of the molecule and may allow *in vivo* tracking of the therapeutic dose and facilitate dosimetry. In this study the synthesis of a fluorescent or radio-labeled Trastuzumab-PEI and *in vitro* uptake, and binding affinity to the Her2/neu expressing human breast adenocarcinoma SKBr3 cells is discussed. In the studies the fluorescent- or ^149^Pm label was synthesized on the polymer, and then the labeled polymer was cross-linked to Trastuzumab.

## 2. Experimental Section

### 2.1. Materials

The University of Missouri Research Reactor (MURR) (Columbia, MO, USA) provided 0.37 GBq (10 mCi) of ^149^Pm in 150 μL of 0.1 M HCl. *S*-2-(4-Isothiocyanatobenzyl)-1,4,7,10-tetraazacyclo-dodecane-tetraacetic acid (DOTA-NCS) was purchased from Macrocyclics (Dallas, TX, USA). Econopack 10DG and Bio Gel 100 size exclusion columns were purchased from Bio-Rad (Bio-Rad Laboratories, Hercules, CA, USA). Centricon^®^ 10K centrifugal filter devices, isotemp economical dry bath incubator, Oakton Acorn pH meter, 2 mL microcentrifuge tubes and 45 µm Nalgene sterile analytical filter units were purchased form Fisher Scientific (Pittsburgh, PA, USA). The centrifuge filter devices were centrifuged on setting 9 with a Fisher Centrific model 225 (Pittsburgh, PA, USA). Oregon Green^®^ 488 carboxylic acid succinimidyl ester was purchased from Invitrogen Corporation (Carlsbad, CA, USA). Traut’s reagent, DMF, Sulfosuccinimidyl 4-[*N*-maleimidomethyl] cyclohexane-1-carboxylate (Sulfo-SMCC), BupH phosphate-buffered saline packs, Slide-A-Lyzer 10K MWCO dialysis cassettes were purchased from Pierce (Rockford, IL, USA). Trastuzumab was obtained from the hospital pharmacy at the University of New Mexico Health Sciences Center (Albuquerque, NM, USA). Polyethylenimine (PEI) with a MW = 60,000 in a 50% solution of water was purchased from Fisher Scientific (Pittsburgh, PA, USA), and all other reagents were purchased Aldrich (Milwaukee, WI, USA), or Sigma (St. Louis, MO, USA). All aqueous solutions were made with 18 MΩ water and were adjusted with either concentrated HCl or 15% NaOH to the desired pH. The buffers were eluted from a column of Chelex 100, the pH measured, and then filtered. Radiolabeling was performed in acid washed 2 mL microcentrifuge tubes. For the ^149^Pm experiments a set of standards was prepared consisting of 0.1 mL of the ^149^Pm solution diluted to 1 mL with HEPES buffer (1 M pH = 6.8). Three subsequent solutions were prepared with a 10-, 100-, and 1000-fold dilution of activity. The efficiency of the Wallace Wizard 1480 automatic gamma counter was determined for ^149^Pm by generating a standard curve of counts per second *versus* disintegrations per second calculated using the calibration of the ^149^Pm shipment. The efficiency was the slope (0.01) of the line and was used to convert sample counts into GBq.

### 2.2. Synthesis of Trastuzumab-Oregon Green^®^ 488 (Trastuzumab-OG)

**Figure 1:**
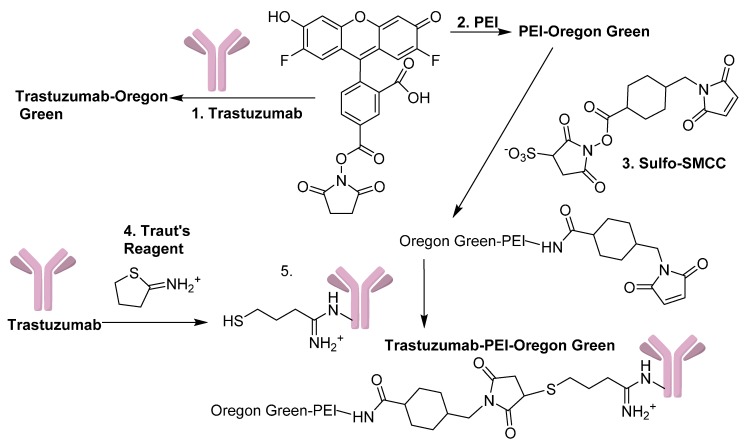
Synthesis scheme used to make PEI-OG, Trastuzumab-OG, and Trastuzumab-PEI-OG. (1) and (2) NaHCO_3_ pH = 8.3, (3) PBS pH = 7.4, 5 h, (4) PBS/0.005 M EDTA (pH = 8.0) 30 min, and (5) PBS/0.005 M EDTA (pH = 7.4) 6 h.

Trastuzumab-Oregon Green^®^ 488 was synthesized according to Figure 1. To a vial placed in an ice bath 1.0 mL of Trastuzumab stock solution was added, and 2 mL of water was added drop wise. Dialysis was performed 2 × 1 L of 0.2 M sodium bicarbonate pH = 8.3, and the sample was concentrated to 1 mL by centrifugation with 10 kDa Centricon^®^ filters devices. Trastuzumab-Oregon Green^®^ 488 was prepared and characterized according to published methods [19], and a 5:1 ratio of Oregon Green^®^ 488 carboxylic acid succinimidyl ester (1.96 × 10^−8^ mol) to Trastuzumab (3.448 × 10^−9^ mol) was used in the reaction. The sample was left overnight and concentrated to 0.5 mL with 10 kDa Centricon^®^ filter device. Purification was performed by centrifugation with 6 × 1.5 mL phosphate-buffered saline (PBS); the sample was concentrate to ~0.5 mL, and the protein concentration and the ratio of Oregon Green^®^ 488 per antibody were determined according to published methods [9].

### 2.3. Synthesis of PEI-Oregon Green^®^ 488 (PEI-OG) 

PEI-Oregon Green^®^ was conjugated, purified, and characterized according to published methods (Figure 1), [9].

### 2.4. Synthesis of Trastuzumab-PEI-Oregon Green^®^ 488 (Trastuzumab-PEI-OG)

Trastuzumab-PEI-Oregon Green^®^ 488 was synthesized according to Figure 1. Sulfo-SMCC (1.97 × 10^−6^ mol) was added to PEI-OG (8.53 × 10^−8^ mol), and the sample was mixed for 5 h, purified with a PD-10 column, and concentrated by centrifugation to 1 mL. A 10 kDa Centricon^®^ filter device was used to change the buffer of the 0.001 g (0.649 mL of a 1.54 mg/mL sample) of Trastuzumab (7.14 × 10^−9^ mol) to PBS/0.005 M EDTA (pH = 8.0), and the sample was concentrated to 0.5 mL. A working solution of Traut’s reagent (3.63 × 10^−4^ M) was prepared and 0.1 mL of Traut’s reagent (3.63 × 10^−8^ mol) was added to the Trastuzumab solution. Purification with a 10 DG column was performed and fractions 3–5 were pooled and added to a centrifuge filter device. A 0.15 mL portion of the modified PEI-OG (1.2795 × 10^−8^ mol) was added to the antibody solution, concentrated to ~0.6 mL, transferred to a vial and mixed for 6 h. A Bio Gel P-100 size exclusion column with a bed volume of 0.8 mL was used to purify the sample, and UV-VIS absorbance at 280 and 496 nm was used to determine the ratio of PEI/Oregon Green^®^ 488/Trastuzumab according to published methods [9,19].

### 2.5. Synthesis and Labeling of DOTA-PEI-Maleimide 

**Figure 2:**
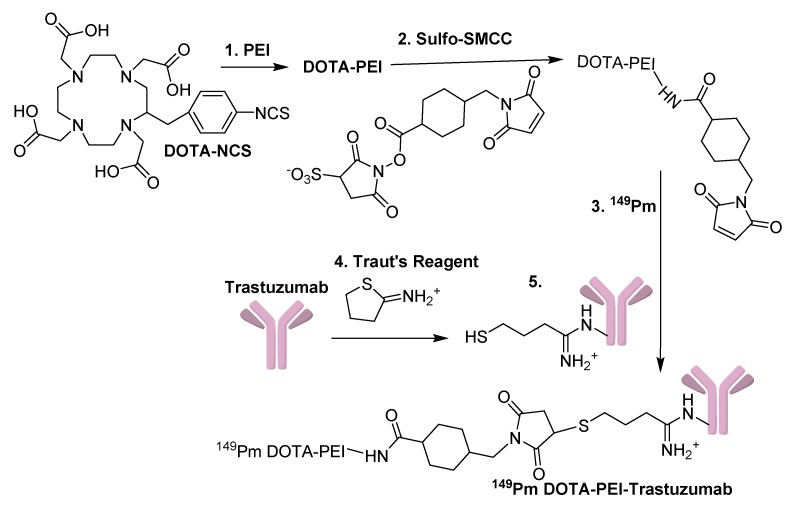
Synthetic scheme used to make ^149^Pm-DOTA-PEI-Trastuzumab. Reaction conditions: (1) NaHCO_3_ pH = 8.5, (2) PBS, RT, 1 h, (3) NH_4_Ac pH = 6.0, 80 °C, 1 h, (4) NaHCO_3_ pH = 8.5, EDTA, RT, 1 h, and (5) PBS, 3 h, RT.

The synthesis and labeling of DOTA-PEI-Maleimide was performed according to Figure 2. DOTA-PEI was prepared according to previously published methods [9] and a mole ratio of 5:1 of DOTA-NCS (7.45 × 10^−6^ mol) to PEI (1.43 × 10^−7^ mol) was used in the reaction. To 3.5 mL of the 2.04 × 10^−5^ M DOTA-PEI (7.15 × 10^−8^ mol) solution was added 0.64 mg of sulfo-SMCC (1.46 × 10^−6^ mol), and the reaction was incubated at RT for 1 h. A 0.5 mL portion of the sample was added to a 10 kDa Centricon^®^ filter devices, diluted to ~2 mL with 1 M NH_4_Ac pH = 6.0 and concentrated to ~0.4 mL. This was repeated three times for the sample, and the 3.5 mL sample was purified with this approach. The samples were pooled into a vial and diluted to 3.5 mL with a final concentration of 2.04 × 10^−5^ M. A 0.05 mL sample of DOTA-PEI-maleimide (1.02 × 10^−9^ mol) was diluted to 1 mL. The UV absorbance at 280 and 290 nm were used to determine the ratio of the chelator to PEI using published methods [20].

### 2.6. Synthesis of Trastuzumab Containing a Thiol Group

The modified Trastuzumab containing a thiol group was prepared according to published procedures [9], and a 3.8 to 1 ratio of Traut’s reagent (7.99 × 10^−9^ mol) to Trastuzumab (2.05 × 10^−9^ mol) was used in the reaction. The sample was purified by centrifugation 2 × 2 mL with PBS/0.005 M EDTA and then concentrated to 0.5 mL with a 10 kDa Centricon^®^ filter.

### 2.7. Synthesis of ^149^Pm-DOTA-PEI-Maleimide

To a vial was added 0.25 mL of 2.03 × 10^−5^ M DOTA-PEI-maleimide (5.11 × 10^−9^ mol) and 0.04 mL ^149^Pm (0.122 GBq). The sample was heated at 80 °C for 1 h, and 56% radiochemical incorporation of the metal was present by TLC (Rf ≤ 0.15 developed with 0.004 M DTPA). The temperature was increased to 90 °C for 1.5 h; the volume was reduced to 0.1 mL, and the incorporation of ^149^Pm was determined by TLC. To the sample was added 0.9 mL of PBS, and the sample was used without purification. 

### 2.8. Synthesis of ^149^Pm-DOTA-PEI-Trastuzumab 

^149^Pm-DOTA-PEI-Trastuzumab was synthesized according to Figure 2. The ^149^Pm-DOTA-PEI-maleimide and the modified Trastuzumab containing a thiol group were combined in a 10 kDa Centricon^®^ size exclusion filter device. The sample was concentrated to ~0.5 mL, and the cross linking reaction was left for 3 h at RT. The sample was purified with a Bio Gel 100 column (bed volume of 0.8 mL). The activity was present in fraction 1 and 2, which were pooled, concentrated with a 10 kDa Centricon^®^ filter device to 0.2 mL, and the radioactivity was determined.

### 2.9. SkBr-3 Cell Studies

To evaluate the HER/2neu receptor binding of the bioconjugates, human breast adenocarcinoma SkBr-3 cells were used. The SkBr-3 cells were obtained from American Type Culture Collection (Manassas, VA, USA) and grown in McCoy’s 5a medium supplemented with 1.5 mM l-glutamine, 10% (*v*/*v*) fetal bovine serum, 10%, 100 units/mL penicillin, and 100 µg/mL streptomycin. The cells were grown at 37 °C, in a humidified atmosphere of 5% CO_2_ and 95% air. The cells were washed with 2 mL PBS and incubated in culture media containing HEPES buffer. SKBR3 cells were put into four-well chamber slides and incubated overnight in 5% CO_2_ incubator at 37 °C at a concentration of 50,000 cells in 0.5 mL media (McCoys 5A media + 10% FBS with l-Glutamine) per well. Media was replaced at the start of the study (0.5 mL) and 0.02 mL of 1 × 10^−6^ M solutions of the fluorescent labeled compounds added to each well. The samples were incubated (37 °C, 5% CO_2_) for 2 and 18 h followed by PBS washes (3×, 0.5 mL). Cells were then fixed with 1.0 mL fresh 3.2% paraformaldehyde solution for 20 min at room temperature in the dark. Fixative was removed and mounting media was applied to the chamber slide. Slides were analyzed using a Confocal LSM10 microscope Zeiss, 1.4NA, 63× objective, on Axioplan 2 microscope and a 488 argon laser. Cells were also analyzed with a differential interference contrast (DIC), polar light.

### 2.10. Radio-Ligand Binding Studies

Approximately 100,000 cells per well were transferred to six-well plates, washed with 2 mL PBS, incubated in culture media containing HEPES buffer and known increasing quantities of ^149^Pm-DOTA-PEI-Trastuzumab. To determine non-specific binding the cells were incubated with excess of Trastuzumab to get a final concentration of 1 µM. After adding the radioligand the cells were incubated for 4 h at 37 °C. The cells were processed as described above, and the radioactivity associated with the final pellet was determined. Graph Pad Prism version 5 (San Diego, CA, USA) was used for saturation binding analysis.

## 3. Results

### 3.1. Fluorescent Compounds and Cell Uptake

**Figure 3:**
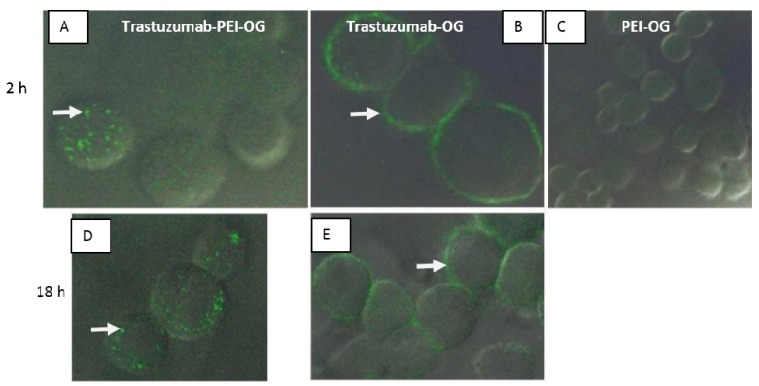
Confocal microscope images of SKBr3 cells incubated for 2 and 18 h with (**A,D**) Trastuzumab-PEI-OG, (**B**,**E**) Trastuzumab-OG, and (**C**) PEI-OG. The Trastuzumab-PEI-OG was visible inside the cell (**A** and **D** arrows), and Trastuzumab-OG was visible around the cells (**B** and **E** arrows).

PEI-OG, Trastuzumab-OG and Trastuzumab-PEI-OG were prepared according to Figure 1, and characterized by UV-VIS absorbance at 280 nm and the 496 nm. A correction factor (CF) was used in the calculations and corresponds to the amount of absorbance of Oregon Green^®^ 488 at 280 nm [9,19]. The ratio of Oregon Green^®^ 488 to Trastuzumab or PEI in Trastuzumab-OG and PEI-OG was 1.69:1 and 5.36:1 respectively. The ratios for the purified Trastuzumab-PEI-OG were 1.04 PEI to 5.57 Oregon Green^®^ 488 to 1 Trastuzumab. At 2 and 18 h Trastuzumab-OG had binding to the surface of the cells with minimal internalization (Figure 3B,E), but Trastuzumab-PEI-OG was internalized into the cells (Figure 3A,D). Incubation of SKBr3 cells with PEI-OG resulted in no cell uptake at 2 h (Figure 3C).

### 3.2. Synthesis of ^149^Pm-DOTA-PEI-Trastuzumab and Cell Binding

**Figure 4:**
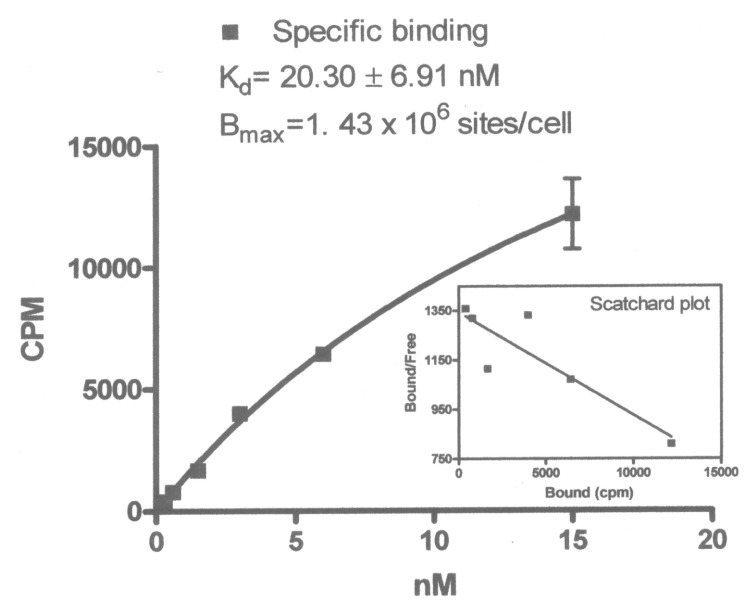
Dissociation constant data (Kd) for ^149^Pm-DOTA-PEI-Trastuzumab with human breast adenocarcinoma SkBr-3 cells (*n* = 3).

**Figure 5:**
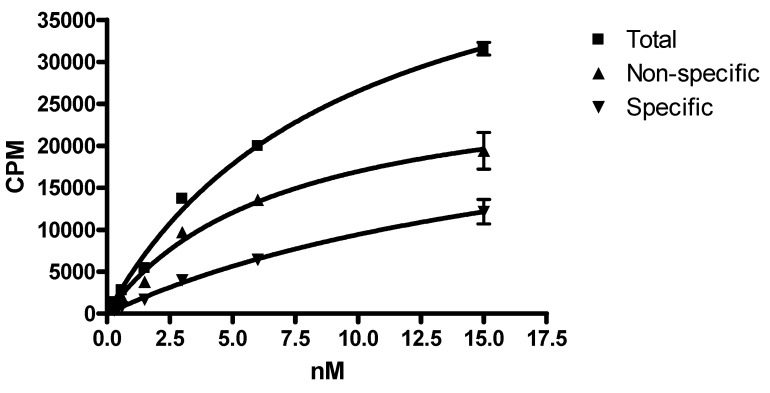
Total, nonspecific and specific binding for ^149^Pm-DOTA-PEI-Trastuzumab with human breast adenocarcinoma SkBr-3 cells (*n* = 3).

DOTA-PEI was synthesized (Figure 2) and UV absorbance at 280 and 290 was used to determine the PEI contained 16.5 chelators (Abs_280_ = 0.11638 and A_290_ = 0.0843). When incubated at 80 °C for 1 h incorporation of ^149^Pm into the DOTA-PEI was 56% by TLC. Increasing the temperature to 90 °C for 1.5 h reduced the volume to 0.1 mL, but did not increase the incorporation of ^149^Pm into DOTA-PEI. After crosslinking to Trastuzumab and purification the radiochemical purity of ^149^Pm-DOTA-PEI-Trastuzumab was determined by TLC (Rf < 0.15 developed with 0.004 M DTPA) to be 96.7%. The radiochemical yield was 28.8% with a specific activity of 0.118 TBq/g, and the total activity associated with ^149^Pm-DOTA-PEI-Trastuzumab was 0.035 GBq. The dissociation constant (Kd) for the ^149^Pm-DOTA-PEI-Trastuzumab for human breast adenocarcinoma SkBr-3 cells was determined to be 20 ± 6.9 nM (Figure 4). Nonspecific binding was higher for ^149^Pm-DOTA-PEI-Trastuzumab than the specific binding to the receptor (Figure 5).

## 4. Discussion

The level of HER2 receptor expression is tested for in breast cancer patients, and the receptor controls aspects of cell growth and division in tumors [21]. Trastuzumab binds to the receptor and can prevent cell growth, this makes the antibody an attractive candidate for targeted therapy. One lysine residue was in the complementarity-determining region at position 64 [22,23]; however, none of the 25 lysine residues were identified as essential for the interaction of Trastuzumab with the HER2/neu receptor [24].

Various synthetic approaches have been utilized to cross-link Trastuzumab to carrier molecules, such as polymers and dendrimers. In one cross-linking approach a homofunctional cross-linker (DSP) was used which could lead to intra or intermolecular cross-linking [25]. Ideally the cross-linker should be heterobifunctional to limit the formation of products other than those desired. The use of *N*-succinimidyl-3-(2-pyridydithio) propionate (SPDP) on both the antibody and the carrier molecule has resulted in a linkage utilizing a disulfide. The use of SPDP as a cross-linker has produced mixed results with PEI and Trastuzumab. Efficient binding and uptake was reported of a Trastuzumab-polyethylenimine(25 kDa)-poly-ethylene glycol conjugate cross-linked with SPDP [26], and antigen-nonspecific EGFR expression was observed with a Trastuzumab-PEI (25 kDa) conjugate [27]. The authors tried cross-linking a PEI with a maleimide group to a monovalent antibody fragment cleaved at the disulfide bridges at the hinge of the heavy chains and resulted in antigen-specific gene delivery. In another approach a dendrimer was cross-linked to Trastuzumab by an SPDP/thiol approach and the molecule showed cellular uptake and internalization similar to the unmodified antibody [28].

The first study in this paper focused on the synthesis of PEI-OG, Trastuzumab-OG, and Trastuzumab-PEI-OG and cell uptake and internalization into SKBr3 cells (Figure 3). The lack of cell binding or cell uptake of PEI-OG into the cells indicates a targeting group is needed. The higher cell internalization of Trastuzumab-PEI-OG compared to Trastuzumab-OG indicates the PEI aids in the cell internalization. The likely sequence of internalization of Trastuzumab-PEI-OG into SKBr3 cells starts with the Trastuzumab portion of the molecule binding to the HER2/neu receptor. The PEI portion of the molecule is a cationic polymer with a large excess of positive surface charge, associates with the plasma membrane of cells. This interaction facilities the endocytosis of the molecule [10].

The high amount of internalization of Trastuzumab-PEI-OG into SKBr3 cells is interesting for applications of alpha radioimmunotherapy. Cell internalization of PEI containing an alpha emitting isotope, such as ^225^Ac, would lead to the radiation being closer to the nucleus of the cell. The proximity of the emitted alpha particles to DNA would cause a higher amount of double stranded DNA breaks and, thus, results in more radiation damage to DNA and an increase in radiotoxicity [6].

The second study in this paper examined a post-labeling approach to synthesize ^149^Pm-DOTA-PEI-Trastuzumab (Figure 2). Loss of the crosslinking ability to maleimide has been reported at pH values greater than 7.5 [29,30]. Therefore the synthetic sequence to prepare DOTA-PEI-maleimide was chosen to reduce degradation of the maleimide groups on the PEI. Linking multiple DOTA chelators to an antibody has limitations because the chelators can interfere with the function of the antibody, but PEI allows multiple chelators to be attached without compromising the integrity of the antibody. The high number of chelates on the PEI should have caused a high incorporation of ^149^Pm into the DOTA, but this was not observed (56%). An increase in temperature and incubation time did not increase the chelation of the ^149^Pm, and similar results were observed in a previous study [8]. Steric interferences of the polymer chain may have decreased the accessibility of DOTA to chelate ^149^Pm. A second issue that could decrease chelation of ^149^Pm is if the four negative charged acid groups on DOTA interact with the positive charges on PEI. This could lead to an orientation of DOTA where chelation of a metal is slower or hindered.

The synthesis approached produced 0.035 GBq of ^149^Pm-DOTA-PEI-Trastuzumab with a specific activity of 0.118 TBq/g and is comparable to a pre-labeling study with ^225^Ac DOTA IgG (0.004 TBq/g) [17]. A pre-labeling study to synthesize ^149^Pm-DOTA-Trastuzumab produced a much lower specific activity (0.000047 TBq/g) [31] then the approach discussed herein to synthesize ^149^Pm-DOTA-PEI-Trastuzumab. The higher amount of DOTA on the PEI and the stability of the DOTA-PEI allowed radiolabeling at higher temperature (80 °C) compared to radiolabeling DOTA-NCS at lower temperatures (56 °C) [9,17,31]. The dissociation constant (Kd) for ^149^Pm-DOTA-PEI-Trastuzumab in human breast adeno-carcinoma SkBr-3 cells was determined to be 20 nM (Figure 4). This is higher than the Kd for Trastuzumab (8–14 nM), but is similar to the Kd for fragments of Trastuzumab (14–36 nM) [32]. Literature studies indicate ^111^In DOTA-Trastuzumab remains bound to the HER2/neu receptor and is not internalized [33]. Cell binding studies with ^149^Pm-DOTA-PEI-Trastuzumab produced very high non-specific binding in human breast adeno-carcinoma SkBr-3 cells (Figure 5). At some concentrations the non-specific binding was approximately two times higher than specific binding. Internalization of ^149^Pm-DOTA-PEI-Trastuzumab into the human breast adeno-carcinoma SkBr-3 cells would account for the high non-specific binding observed in the cell studies. This is consistent with the internalization of Trastuzumab-PEI-OG in human breast adeno-carcinoma SkBr-3 cells. The cytotoxicity of ^149^Pm-DOTA-PEI-Trastuzumab to SkBr-3 cells will be examined in future studies.

## 5. Conclusions

This research examined the synthesis of Trastuzumab-PEI-OG, PEI-OG, Trastuzumab-OG, ^149^Pm-DOTA-PEI-Trastuzumab and subsequent *in vitro* studies with Her2/neu expressed on human breast adenocarcinoma SkBr-3 cells. The internalization of the Trastuzumab-PEI-OG into SkBr-3 cells is an important finding for radioimmunotherapy as radiolabeling the compound with therapeutic isotopes could result in higher radiation damage and a higher toxicity. Cell binding data of ^149^Pm-DOTA-PEI-Trastuzumab indicates the compound has high affinity for human breast adenocarcinoma SkBr-3 cells. These findings and the retention of ^225^Ac and daughters by the PEI in our previous published study [8] indicate DOTA-PEI-Trastuzumab has potential as a carrier of ^225^Ac and ^149^Pm to tumors.

## References

[1] National Research Council (US) and Institute of Medicine (US) Committee on State of the Science of Nuclear Medicine (2007). Targeted Radionuclide Therapy. Advancing Nuclear Medicine through Innovation.

[2] Slamon D.J., Clark G.M., Wong S.G., McGuire W.L. (1987). Human breast cancer: Correlation of relapse and survival with amplification of the HER-2/neu oncogene. Science.

[3] Berchuck A., Kamel A., Whitaker R., Kerns B., Olt G., Kinney R., Soper J.T., Dodge R., Clarke-Pearson D.L., Marks P. (1991). Overexpression of HER-2/neu is associated with poor survival in advanced epithelial ovarian cancer. Cancer Res..

[4] Carlsson J., Nordgren H., Sjöström J., Wester K., Villman K., Bengtsson N.O., Ostenstad B., Lundqvist H., Blomqvist C. (2004). HER2 expression in breast cancer primary tumors and corresponding metastases. Original data and literature review. Br. J. Cancer.

[5] Le X., Pruefer F., Bast R. (2005). HER2-targeting antibodies modulate the cyclin-dependent kinase inhibitor p27Kip1 via multiple signaling pathways. Cell Cycle.

[6] McDevitt M.R., Scheinberg D.A. (2002). Ac-225 and her daughters: the many faces of Shiva. Cell Death Differ..

[7] Fritzberg A.R. (1998). Antibody pretargeted radiotherapy: A new approach and a second chance. J. Nucl. Med..

[8] Fitzsimmons J., Atcher R. (2007). Synthesis and evaluation of water-soluble polymer to reduce Ac-225 daughter migration. J. Label. Compd. Radiopharm..

[9] Fitzsimmons J., Atcher R., Cutler C. (2015). Development of a prelabeling approach for a targeted nanochelator. J. Radioanal. Nucl. Chem..

[10] Bieber T., Meissner W., Kostin S., Niemann A., Elsasser H. (2002). Intracellular route and transcriptional competence of polyethylenimine–DNA complexes. J. Control. Release.

[11] Sonawane N., Szoka F., Verkman A. (2003). Chloride accumulation and swelling in endosomes enhances DNA transfer by polyamine-DNA polyplexes. J. Biol. Chem..

[12] Barr M., Atcher R. (2005). Personal communication.

[13] Peng L., Gao Y., Xue Y., Huang S., Zhou R. (2013). The effectiveness, cytotoxicity, and intracellular trafficking of nonviral vectors for gene delivery to bone mesenchymal stem cells. J. Bioact. Compat. Polym..

[14] Yuan T., Wang Y., Cao W., Sun Y., Liang J., Fan Y., Zhang X. (2014). Reducible cationic PAA-*g*-PEI polymeric micelle/DNA complexes for enhanced gene delivery. J. Bioact. Compat. Polym..

[15] Clinical Trial—Targeted Atomic Nano-Generators in Patients with Advanced Myeloid Malignancies. https://clinicaltrials.gov/ct2/show/NCT00672165.

[16] INCHEM IPCS Report: Polyethylenimine and Ethylenimine. http://www.inchem.org/documents/jecfa/jecmono/v20je08.htm.

[17] McDevitt M., Scheinberg D.A., Simon J., Frank R.K., Scheinberg D.A. (2002). Design and synthesis of ^225^Ac radioimmuno-pharmaceuticals. Appl. Radiat. Isot..

[18] Hu F., Cutler C., Hoffman T., Sieckman G., Volkert W., Jurisson S. (2002). Pm-149 DOTA bombesin analogs for potential radiotherapy. *In vivo* comparison with Sm-153 and Lu-177 labeled DO3A-amide-βAla-BBN(7–14)NH_2_. Nucl. Med. Bio..

[19] ThermoFisher Amine-Reactive Probes. https://tools.thermofisher.com/content/sfs/manuals/mp00143.pdf.

[20] Fitzsimmons J. (2005). The synthesis and characterization of metal complexes for imaging and therapy. Ph.D. Thesis.

[21] Goodell V., Waisman J., Salazar L.G., de la Rosa C., Link J., Coveler A.L., Childs J.S., Fintak P.A., Higgins D.M., Disis M.L. (2008). Level of Her-2/neu protein expression in breast cancer may affect the development of endogenous Her-2/neu specific immunity. Mol. Cancer Ther..

[22] Carter P., Presta L., Gorman C.M., Ridgway J.B., Henner D., Wong W.L., Rowland A.M., Kotts C., Carver M.E., Shepard H.M. (1992). Humanization of an anti-p185HER2 antibody for human cancer therapy. Proc. Natl. Acad. Sci. USA.

[23] Cho H.S., Mason K., Ramyar K.X., Stanley A.M., Gabelli S.B., Denney D.W., Leahy D.J. (2003). Structure of the extracellular region of HER2 alone and in complex with the Herceptin Fab. Nature.

[24] Kelley R.F., O’Connell M.P. (1993). Thermodynamic analysis of an antibody functional epitope. Biochemistry.

[25] Chiu S., Ueno N., Lee R. (2004). Tumor-targeted gene delivery via anti-HER2 antibody (trastuzumab, Herceptin) conjugated polyethylenimine. J. Control. Release.

[26] Germershaus O., Merdan T., Bakowsky U., Behe M., Kissel T. (2006). Trastuzumab-polyethylenimine-polyethylene glycol conjugates for targeting Her2-expressing tumors. Bioconjugate Chem..

[27] Strehblow C., Schuster M., Moritz T., Kirch H.C., Opalka B., Petri J.B. (2005). Monoclonal antibody–polyethyleneimine conjugates targeting Her-2/*neu* or CD90 allow cell type-specific nonviral gene delivery. J. Control. Release.

[28] Shukla R., Thomas T.P., Peters J.L., Desai A.M., Kukowska-Latallo J., Patri A.K., Kotlyar A., Baker J. (2006). HER2 specific tumor targeting with dendrimer conjugated anti-HER2 mAb. Bioconjugate Chem..

[29] ThermoScientific Instruction Manual for Sulfo-SMCC and SMCC. https://tools.thermofisher.com/content/sfs/manuals/MAN0011295_SMCC_SulfoSMCC_UG.pdf.

[30] Pierce bulletin Instructions BMPH, EMCH and KMUH. https://www.funakoshi.co.jp/data/datasheet/PCC/22106.pdf.

[31] Fitzsimmons J., Nayak T., Cutler C., Atcher R. (2008). Synthetic development of Herceptin bioconjugates for targeting tumors with ^149^Promethium. J. Nucl. Med..

[32] Tang Y., Wang J., Scollard D.A., Mondal H., Holloway C., Kahn H.J., Reilly R. (2005). Imaging of HER2/neu-positive BT-474 human breast cancer xenografts in athymic mice using ^111^In-trastuzumab (Herceptin) Fab fragments. Nucl. Med. Biol..

[33] Constantini D., Chan C., Cai Z., Vallis K., Reilly R. (2007). ^111^In-Labeled Trastuzumab (Herceptin) Modified with Nuclear Localization Sequences (NLS): An Auger Electron-Emitting Radiotherapeutic Agent for HER2/neu-Amplified Breast Cancer. J. Nucl. Med..

